# Partnership research on nutrition transition and chronic diseases in West Africa – trends, outcomes and impacts

**DOI:** 10.1186/1472-698X-11-S2-S10

**Published:** 2011-11-08

**Authors:** Hélène Delisle, Victoire Agueh, Benjamin Fayomi

**Affiliations:** 1 TRANSNUT, WHO Collaborating Centre on Nutrition Changes and Development, Department of Nutrition, University of Montreal, P.O. Box 6128, Station Main, Montreal, QC H3C 3J7, Canada; 2 Institut régional de santé publique (IRSP) [Regional Institute of Public Health], Ouidah, Benin, West Africa; 3 Institut des sciences biomédicales appliqués (ISBA) [Applied Biomedical Science Institute], Cotonou, Benin, West Africa

## Abstract

**Background:**

Nutrition-related chronic diseases (NRCD) are rising quickly in developing countries, and the nutrition transition is a major contributor. Low-income countries have not been spared. Health issues related to nutritional deficiencies also persist, creating a double burden of malnutrition (DBM). There is still a major shortage of data on NRCD and DBM in Sub-Saharan Africa. A research program has been designed and conducted in partnership with West African institutions since 2003 to determine how the nutrition transition relates to NRCD and the DBM in order to support prevention efforts.

**Methods:**

In Benin, cross-sectional studies among apparently healthy adults (n=540) from urban, semi-urban and rural areas have examined cardiometabolic risk (hypertension, obesity, dyslipidemia, insulin resistance) in relation to diet and lifestyle, also factoring in socio-economic status (SES). Those studies were followed by a longitudinal study on how risk evolves, opening the way for mutual aid groups to develop a prevention strategy within an action research framework. In Burkina Faso, a cross-sectional study on the nutritional status and dietary patterns of urban school-age children (n=650) represented the initial stages of an action research project to prevent DBM in schools. A cross-sectional study among adults (n=330) from the capital of Burkina Faso explored the coexistence, within these individuals, of cardiometabolic risk factors and nutritional deficiencies (anemia, vitamin A deficiency, chronic energy deficiency), as they relate to diet, lifestyle and SES.

**Results:**

The studies have shown that the prevalence of NRCD is high among the poor, thereby exacerbating social inequalities. The hypothesis of a positive socio-economic (and rural–urban) gradient was confirmed only for obesity, whereas the prevalence of hypertension, insulin resistance and dyslipidemia did not prove to be higher among affluent city dwellers. Women were particularly affected by abdominal obesity, at 48% compared to 6% of men. Protective factors against the risk of NRCD were physical activity and adequate micronutrient intake. The research also showed that nutritional deficiencies were not restricted to schoolchildren in rural areas because in the capital of Ouagadougou, for example, 40% of schoolchildren were anaemic and 40% were vitamin A deficient. Partnership research has expanded to include advocacy and human resources training.

**Conclusion:**

These initial studies on NRCD in West Africa indicate the relevance and urgency of prevention, even among low-income groups and countries. They show that the fight against NRCD as well as nutritional deficiencies should focus on women. Seeing how researchers from the African partner institutions have connections with decision-making authorities, the research findings could have an impact on prevention policies and programs in communities and schools alike. Greater support must nevertheless be provided to lobbying and advocacy work for an even greater impact. As well, the sustainability of the research program remains a challenge that requires resource mobilization and training for the purpose.

## Introduction

### Chronic diseases are affecting low-income countries

Nutrition-related chronic or non-communicable diseases (NRCD) — mainly diabetes and cardiovascular diseases (CVD) — have been deemed a priority by the WHO [1]. These diseases are continually on the rise worldwide, but particularly in developing countries, as a result not only of ageing populations, but also of increasingly Westernized diets and lifestyles. The international community has been sounding the alarm since 2005 [2], but these diseases continue to be neglected in developing countries, where they are harming individual health and burdening health care systems. Chronic diseases are deadlier in developing countries than in industrialized ones, and the discrepancy is even greater for women than men [3]. Evidence is needed regarding NRCD and their determinants in low-income developing countries, although the lack of data is not the only obstacle preventing decision-makers from taking appropriate action [4]. "North–South" partnership research can simultaneously enhance knowledge and promote the application of findings.

### The double burden of malnutrition increases health-related inequalities

The emergence of these "civilization" or lifestyle diseases unfortunately does not mean that undernutrition and micronutrient deficiencies have disappeared. On the contrary, the latter continue to be responsible for high morbi-mortality rates among women and children, particularly in low-income developing countries. This "double burden of malnutrition" (DBM), resulting from the coexistence of diseases of deficiency and excess, poses a double challenge [5]. Women are particularly affected, which further exposes the inequalities between men and women [6,7]. An additional source of concern is that, according to the theory of developmental origins of chronic diseases, fetal or childhood malnutrition increases the risk of nutrition-related chronic diseases in adulthood [8]. Thus, the two types of malnutrition go hand in hand, again exacerbating the chronic disease risk associated with nutrition transition [6].

### Need for a better understanding of nutrition transition

Nutrition transition, as well as its determinants, characteristics and incidence is at the heart of the research described below. Our goal is to document the dietary patterns and food intake of urban populations in West Africa to promote an adequate and preventive diet adapted to the socio-cultural and economic environment. Diet is one of many lifestyle factors — such as physical activity or alcohol and tobacco use [9] — that can either prevent or increase the risk of several chronic diseases, but it is one that is often overlooked.

## Methodology

### Overview

This is more of an evolving research program in West Africa than a one-time project, the advantage being to illustrate the progress being made in the type of research, impacts and partnership. The various stages of the research process are presented, including efforts made to integrate findings into policies and programs, as well as the generation of new research questions and assumptions [10]. Our studies on nutrition transition and the DBM — conducted since 2003 in partnership with researchers from Benin and, more recently, Burkina Faso and other countries from the subregion of Francophone West Africa — are the first to be conducted on this issue in low-income African countries. An exploratory study on the double burden of malnutrition was first undertaken among urban households in Cotonou, the economic centre of Benin. Cross-sectional studies on nutrition transition and the cardiometabolic risk factors were then conducted in Cotonou, in a small town and in a rural area of Benin until 2008. These were followed by cross-sectional studies on DBM in Burkina Faso. Preventive action research initiatives were then implemented in schools (Benin, Burkina Faso) and communities (Benin).

### Exploratory study on the incidence of the double burden of malnutrition (DBM) in poor urban households in Cotonou (Benin)

The joint projects in Benin began in 2003 with an exploratory study on the prevalence of DBM. In collaboration with *Institut des sciences biomédicales appliqués* (ISBA), the study was conducted in about 100 households selected at random in the poorer areas of Cotonou [11]. This double burden, linked to a rapid nutrition transition [12], had yet to be investigated in an urban West African environment. The households selected for the study had to include the biological mother and at least two children under 12 years of age. The aim was to determine the prevalence of DBM in an underprivileged urban area — specifically, the occurrence of overweight/obesity in the mother and under-nutrition in the child — and to identify the variables associated with this double burden. The assumption was that a significant proportion of these "double-burden" households would be found, even in poor settings.

### Cross-sectional studies on characteristics and factors associated with nutrition transition in Benin

The main objective of research projects conducted in Benin until around 2008 was to better document nutrition transition and its link to cardiometabolic risk factors. The studies were conducted among apparently healthy adults and were designed to explore the relationship between practices and risks, with a view to helping prevent these diseases by examining lifestyle differences between men and women, as well as differences in socio-economic status (SES) and place of residence. Similar cross-sectional epidemiological studies were conducted in Cotonou [13], Port-au-Prince (Haiti) and among the Haitian community of Montreal [14] to highlight the vastly different risk profiles based on the highly contrasting environments of populations with a common genetic heritage. We will focus, however, on the studies conducted in Benin.

The Benin studies were based on random samples of men and women aged 25 to 60 with no prior diagnosis of diabetes, hypertension or heart disease; they were conducted in the city of Cotonou, in the medium-sized city of Ouidah, and in rural villages surrounding Ouidah for a total of 540 participants, 50% of whom were women [13,15-17]. The assumptions included an expectation of seeing a positive socio-economic and rural–urban gradient for obesity and other cardiometabolic risk factors (hypertension, dyslipidemia, hyperglycemia), and it was expected that women would be more affected than men by obesity and co-morbidity. It was also expected that the cardiometabolic risk factors would be linked to a more Westernized diet and lifestyle. The Benin partner institutions were the ISBA and the *Institut régional de santé publique* (IRSP).

The main biological parameters used to detect cardiometabolic risk were blood pressure, obesity as determined by the body mass index (BMI), abdominal obesity as determined by waist circumference, blood lipids (total cholesterol, HDL cholesterol, triglycerides) and fasting insulin and blood glucose levels. To assess food intake and physical activity, two or three 24-hour recalls were conducted at least one week apart. Two scores were calculated for diet quality: an intake adequacy score for 14 micronutrients based on FAO/WHO recommended intake levels [18]; and a preventive diet score based on eight WHO dietary recommendations for preventing chronic diseases [19]. In addition to age, gender and place of residence, the SES variables were level of education and a score for household amenities, used as a proxy for income. The tertiles for this amenity score were calculated separately for the three areas (large city, small town, rural environment) and were used in the data analyses to define three income levels: low, medium and high. Further details are provided in other publications [15-17].

### Cross-sectional studies on the double burden of malnutrition in schoolchildren and adults in Burkina Faso

This DBM is observed at the population, household and even individual levels [20]. The latter would be a case, for example, of a woman who is obese and anemic or of a man who presents with both emaciation and hypertension. Two studies were conducted in Ouagadougou (Burkina Faso) to examine this issue: one in a school setting, in collaboration with an NGO, Helen Keller International (HKI); the other among a sample of apparently healthy adults, in partnership with the *Institut de recherche en sciences de la santé* (IRSS) [Health Sciences Research Institute] and the *Institut supérieur en sciences de la population* (ISSP) [Higher Institute of Population Sciences].

The goal of the study among schoolchildren was to assess nutritional status, as well as perceptions and dietary (and health) patterns that could foster deficiencies as well as the diseases of excess. It was also used to establish a baseline prior to implementation of the Nutrition-Friendly School Initiative, an intervention framework proposed by WHO and its partners for DBM prevention [21]. The Ministry of Primary Education helped select six primary schools to achieve a balance of secular and denominational, public and private, and urban and peri-urban schools. These six schools were then matched with six other schools, which served as the control group. More than 700 schoolchildren from 12 schools participated in the study. Entire classrooms were selected from two middle-grade (4-5) classes, because this approach was socially more acceptable than selecting individual children and because children of that age (11 and up) could answer questions about their attitudes and behaviours themselves. The weight, height and blood pressure of every child were measured; hemoglobin levels were also measured from a drop of capillary blood (HemoCue^®^ method). Palpation of the thyroid gland was performed to detect goitre. The consumption frequency of predetermined foods identified as either "healthy food" or "junk food" and student perceptions about those foods were explored in a self-administered in-class questionnaire. The SES indicators used were parents' main occupation, perceived food security and pocket money. In a random sub-sample of some 200 schoolchildren, a sample of venous blood was taken to measure serum retinol, as an indicator of vitamin A status, and to measure blood lipids.

The goal of the cross-sectional DBM study recently conducted among adults in Ouagadougou was to assess the occurrence of different double-burden phenotypes within this population. The assumptions to be tested were a higher prevalence of DBM in women than in men, and the predominance of the phenotype of overweight/obesity combined with micronutrient(s) deficiency. A total of 350 subjects (50% women) were selected by stratified random sampling, according to SES, as determined by an index of household amenities. These individuals, aged 25 to 60, had no prior diagnosis of diabetes, hypertension or heart disease. The same biological parameters used in Benin to measure risk of chronic diseases were also used in Burkina Faso. Diet and lifestyle were also examined according to the same parameters. Serum retinol and hemoglobin were measured as well.

## Results

### The double burden of malnutrition found in poor urban environments

The exploratory study conducted in poorer neighbourhoods of Benin's largest city, Cotonou, revealed that in 16% of households, the mothers were overweight or obese and at least one child under the age of 12 presented with chronic or acute malnutrition [11]. Of particular note, a poorly diversified diet was associated with a higher likelihood of DBM, suggesting that food insecurity in terms of quality — itself linked to poverty — could be equally responsible for obesity in mothers and undernutrition in children. Also worthy of note is that maternal overweight, without child undernutrition, was found in 21% of households studied, further highlighting the threat that obesity poses to women.

### Cardiometabolic risk factors: obesity affecting primarily women

The cross-sectional studies conducted in three areas of Benin revealed a positive gradient (from 4.1% to 11%) in the prevalence of metabolic syndrome extending from the rural environment to the highly urbanized environment of Cotonou [15,16]. However, this gradient was found only for obesity and not for the other components of the metabolic syndrome: hypertension, hyperglycemia and dyslipidemia. For example, the prevalence rate of abdominal obesity was 28.2% in rural areas and 52.5% in metropolitan areas. Women were particularly affected, with an obesity rate three times greater than that of men [16]. The difference between men and women was particularly pronounced in urban areas, where women were physically less active than men [15] in the sense that, even though women spent more hours on productive and domestic tasks than men, these non-agricultural tasks required a lesser expenditure of energy. Besides a sedentary lifestyle, SES was also directly and independently correlated with obesity [16]. Physical activity turned out to be a protective factor against not only obesity but also hypertension.

### An adequate supply of micronutrients can reduce the risk of NRCD

Nutrition patterns do not yet seem to be very Westernized in Benin, even in Cotonou [17]. Although we were unable to demonstrate a significant link between diet and obesity, we did detect a positive relationship between better micronutrient intake and lower blood pressure or higher insulin sensitivity (unpublished data). These findings are new because the increased cardiometabolic risk associated not only with overeating, but also with a diet that is lacking in certain micronutrients, had never before been reported in developing countries. Therefore, the fight against nutritional deficiencies could help prevent NRCD as well as nutritional deficiencies. The cross-sectional study on the DBM in adults from Ouagadougou — the data for which is still being analyzed — will help determine whether the relationship between micronutrient intake and biomarkers of cardiometabolic risk can be confirmed.

### Urban schoolchildren are also experiencing micronutrient deficiencies

The cross-sectional study conducted in Ouagadougou on the nutritional status of schoolchildren revealed, in particular, that even in an urban environment, micronutrient deficiencies were widespread. In fact, over 40% of students presented with a vitamin A deficiency and as many presented with iron deficiency anemia, with no significant difference between boys and girls [22]. The prevalence of thinness was proportionally higher in peri-urban than in urban areas. Furthermore, overweight was nearly nonexistent except in private schools. Results were released to the stakeholders, including teachers, parents, administrators and government authorities.

## Discussion

Because this was a program rather than a research project, with numerous studies conducted in partnership in Benin and, to a lesser extent, Burkina Faso, this discussion will cover the trends and impacts of the research rather than the results, per se, which are discussed elsewhere [15-17,22].

### Research trends and impacts

Based on epidemiological studies conducted in Benin, the community action research began around 2008 with a view to preventing NRCD by acting on the risk factors that the studies had more clearly characterized. Self-help groups were, therefore, formed with participants from the cross-sectional studies in the three areas (Cotonou, Ouidah and surrounding rural areas), as a prevention strategy. This was an innovative approach in that, for the first time in Africa, the self-help model was used to prevent chronic diseases. Incidentally, there are few examples, even in industrialized countries, of this strategy being applied to prevent CVD [23].

Furthermore, the WHO Nutrition-Friendly School Initiative [21] — specifically designed to target DBM through health promotion — is currently being tested in pilot schools in Ouagadougou and Cotonou. The cross-sectional study on nutrition in Ouagadougou schoolchildren, as previously mentioned, will allow for an impact assessment to be performed after four years of intervention, because half of the schools under study must serve as the control group against which the comparison can be made. Baseline data from the study was used to identify areas of intervention and to advocate for nutrition-related intervention in urban schools, as will be seen later on. Furthermore, in both Ouagadougou and Cotonou, processes will be assessed in participating schools after two years of operations in order to determine whether the approach is working and to modify any aspects of the intervention, where needed.

Action research in community and school environments, in turn, has stimulated research and development (R&D) aimed at developing appropriate tools for raising the awareness of, and guiding and educating the public. An obvious lack of educational tools for giving adults relevant and consistent advice on diet, physical activity and alcohol became apparent. In partnership with the stakeholders, new initiatives have therefore been undertaken to develop a "food guide" [24], first in Benin and mainly for city dwellers, who have more food choices than rural inhabitants. This food guide has two components: (1) broader recommendations, adapted from WHO dietary recommendations for health [19] and validated locally with various groups of adults; and (2) a pictorial representation of the food groups and number of recommended servings to meet the nutritional needs of different age groups (excluding children under age two). Flip charts and a video depicting general recommendations were produced. The picture boxes are used during self-help group meetings, and the 15-minute video is regularly broadcast on national TV. As for the second component of the Benin food guide, studies are underway to consult the public on how foods should be grouped together; serving sizes will then be defined based on food consumption data obtained from the cross-sectional studies. A simple nutritional risk score is also being developed for use in schools; the baseline data from the study of Ouagadougou schoolchildren is being used to develop this score. The score will then be tested in both Ouagadougou and Cotonou. It will help identify schoolchildren who are at risk and who should undergo a more in-depth nutritional assessment at the individual and family levels. Lastly, a tool is being developed to help advocate for policies and programs designed to prevent and address diabetes. This tool is based on the PROFILES approach [25], which has been a successful advocacy tool in the fight against malnutrition. Its adaptation to NRCD was considered promising by the West African partners. Diabetes was chosen over obesity because it is a known and feared health issue. Obesity, on the other hand, has yet to be perceived as a serious health problem, mainly because of cultural factors that tend to cast overweight in a positive light [26].

The global health research field owes it to itself to not only generate new knowledge and information but also to contribute to a population's well-being [10]. The research program on NRCD and the DBM was designed to have just such an impact, even though this impact cannot be measured at this time. However, based on certain indicators, we can conclude that the research has had a positive influence on intervention programs and policies. The results have been widely disseminated among communities, researchers and decision-makers from Benin and Burkina Faso, as well as the international nutrition and development community, thereby helping to raise awareness among stakeholders. We believe that, because of our work, at least partly, NRCD are being taken into account in Benin's 2007–2016 National Health Development Plan. The creation of a new school of nutrition by the Rector of Benin's University of Abomey-Calavi (UAC) to accommodate the new training program may also be considered an outgrowth of partnership research on nutrition. The release of the Ouagadougou schoolchildren research results in a media event also seems to have had a certain impact, because primary education department in Burkina Faso is now considering introducing school lunch and nutrition programs not only in rural schools but also in urban schools. Lastly, after results from the cross-sectional studies on nutrition transition and NRCD in Benin were disseminated, the public itself expressed the need for information about food choices, which also shows an increase in awareness about these issues.

The next step for partnership research in Benin and Burkina Faso should be to conduct operational research to assess the cost effectiveness of interventions to address and prevent diabetes.

### Evolving partnership

Between 2003 and 2008, TRANSNUT, an interdisciplinary team from the University of Montreal, conducted joint research on NRCD and DBM in Benin with IRSP, an institute of higher learning, and ISBA, a research institute. In Burkina Faso, a research partnership has also been in place for several years with IRSS [Health Sciences Research Institute] and the French *Institut de recherche pour le développement* (IRD) [Research Institute for Development]; the fight against vitamin A deficiency was the main research topic until the partnership expanded to include the DBM in 2008. Former African graduates of University of Montreal advanced studies in nutrition became partners. We have also invested heavily, and continue to do so — for the sake of the research — in students who are enrolled in the nutrition doctoral program at the University of Montreal and who are seconded from partner institutions in Africa because they can also develop research skills in their own countries and strengthen the capacity of their own institutions.

Beyond research, pressing needs were identified with respect to training new human resources in nutrition in order to prevent NRCD and more effectively address DBM. Similarly, it was also determined that a strong advocacy was needed. This is how the partnership — originally limited to research — expanded. In 2008, under CIDA's University Partnerships for Cooperation and Development (UPCD) program, this expanded partnership officially took shape, branched out and was given access to greater resources. In addition to the research component, the six-year project includes "human resources training" and "communication and advocacy" components. The partnership has expanded to include other academic institutions from Benin (University of Abomey-Calavi) and Burkina Faso (University of Ouagadougou), as well as various WHO structures outside Geneva (Regional Office for Africa in Brazzaville, Inter-country Support Team in Ouagadougou, and country representatives in Benin and Burkina Faso). HKI, the NGO providing nutrition-related technical support, also became an official partner in Burkina Faso.

New university nutrition programs have been developed in Benin for the region, drawing on the comparative advantage of Quebec universities in this field. This alliance with universities in French-speaking Africa could help them catch up to their counterparts in English-speaking countries. A new master's program in public health nutrition has been created at the IRSP, and a professional B. Sc. program in nutrition and dietetics is now offered at *Université d’Abomey-Calavi* (UAC), also in Benin. Modules for continuing education in nutrition are also offered through partners from Benin and Burkina Faso. With respect to communication and advocacy, these were discussed above specifically in terms of R&D for the development of appropriate intervention tools. Researchers from partner institutions also play an important decision-making role in the government, such that there is no need to separate "researchers" from "decision-makers." This makes the advocacy work easier and enhances the impact of research on policies and programs.

### Limitations and challenges of partnership research in West Africa

Global health research partnerships experience numerous challenges and limitations, many of which are not specific to the nutrition field and are therefore discussed at length elsewhere [28,29]. In this section, we will discuss limitations or challenges based on our own experience of partnership research in nutrition with institutions from Francophone West Africa.

Although this is a widely known problem, it is worth mentioning that the loss of young researchers, once they have been trained, to NGOs or international organizations that can offer them better living conditions is a major obstacle to strengthening a country's research capabilities. Overcoming this obstacle is therefore one of the goals of partnership research, since research is an essential development tool [30]. Yet it is better for young researchers to continue working within development structures rather than settle in industrialized countries. Counting only the Ph.D. graduates- (in nutrition) from the University of Montreal since 2004 who are originally from Benin or Burkina Faso, two out of six have not returned to their country of origin, although they are working for the development cause in the field of nutrition. Nevertheless, innovative strategies are needed to retain researchers in their home countries.

A major challenge to action research is sustaining local interventions connected with the research once external funding has ended. In this type of research interventions are usually funded by the research budget, and the termination of research funding often means an end to the intervention itself. For example, the self-help groups' NRCD prevention strategy, as implemented in Benin following the epidemiological study, is struggling to survive. The groups will still require an outside facilitator until they can become completely independent, and even then they will need to resort to outside financial and technical resources for screening and advice. The Benin research partners are currently trying to access domestic resources to cover these expenses. Prospects for the sustainability of the Nutrition-Friendly School Initiative may be better because the action research essentially provides technical support and assistance with the implementation process, rather than funding priority activities defined by school nutrition committees, that would have to be funded from schools’ own resources.

To date, ethnocultural issues and the different life-courses of men and women as they relate to nutrition transition and the implications of NRCD and DBM have not been adequately addressed in our research activities. Qualitative research is needed to bring these issues to light. For example, the perceptions that women have of their body image and of the changing urban diet, psychosocial barriers to physical activity and their need for health- and nutrition-related information are only a few of the research topics that deserve further investigation. In addition, many other avenues of research, both quantitative and qualitative, deserve to be explored to provide greater insight into the link between globalization, nutrition transition and NRCD [29]. This would help decision-makers take the type of actions that would curb the potentially deleterious effects of this on nutrition and health.

Lastly, the language issue cannot be ignored. Indeed, for research initiatives to have a greater scope and impact, they must achieve sufficient reach in the scientific literature, which is primarily in English. Furthermore, partners would see their actions strengthened and their influence expanded if they were to join forces with existing networks for the prevention and control of non-communicable diseases such as the Consortium for Non-Communicable Disease Prevention and Control in Africa. More and more people in the countries of Francophone Africa are learning English but those countries still have a long way to go to bridge the gap between them and the English-speaking world.

## Conclusion

This case study presents an example of global health research as a process that aims not only to enhance knowledge and generate new data but also to improve health and reduce inequalities with respect to chronic diseases. It illustrates the link between research, policies and programs, and the training of human resources — a link that is essential but one that also requires strengthening. The initial research partnership has expanded to encompass training and advocacy as well. The institutional strengthening, based on human resources, is a measure of the progressive local ownership of training and nutrition research. As advocacy strategies, linking decision-makers with the research and disseminating and translating the results proved to have a beneficial impact. However, it must be pointed out that lobbying and media mobilization, both locally and internationally, need to be strengthened to have a greater impact on research, training and advocacy efforts targeting NRCD and DBM. With respect to research, the African partners, with a view to appropriating and strengthening the initiatives, now plan to come together around a common research project on the same topic and raise the necessary funds themselves. Training in resource mobilization will be indispensible to the sustainability of the research partnership.

## List of abbreviations used

CIDA: Canadian International Development Agency; CIHR: Canadian Institutes of Health Research; CVD: cardiovascular diseases; DBM: double burden of malnutrition; FAO: Food and Agriculture Organization of the United Nations; IRD: Institut de recherche pour le développement [French Research Institute for Development]; IRSP: Institut régional de santé publique [Benin Regional Public Health Institute]; IRSS: Institut de recherche en sciences de la santé [Burkina Faso Health Sciences Research Institute]; ISBA: Institut des sciences biomédicales appliquées [Benin Applied Biomedical Sciences Institute]; ISSP: Institut supérieur en sciences de la population [Burkina Faso Higher Institute of Population Sciences]; NGO: non-governmental organization; NRCD: nutrition-related chronic diseases; R&D: research and development; SES: socio-economic status; UAC: University of Abomey-Calavi (Benin); WHO: World Health Organization.

## Competing interests

The authors stated that they have no conflict of interest.

## Authors' contributions

All three authors were actively involved in the research program discussed in this article, in terms of study design, data collection and interpretation of results. HD wrote the manuscript. VA and BF provided their comments on the initial version of the case study. They reviewed and approved this manuscript prior to its publication.
